# Editorial: Health-Related Complications of Acromegaly

**DOI:** 10.3389/fendo.2020.00496

**Published:** 2020-07-29

**Authors:** Marek Bolanowski, Cesar L. Boguszewski, Annamaria Colao, Aleksandra Jawiarczyk-Przybyłowska

**Affiliations:** ^1^Department of Endocrinology, Diabetes and Isotope Therapy, Wroclaw Medical University, Wrocław, Poland; ^2^Endocrine Division (SEMPR), Department of Internal Medicine, Federal University of Paraná, Curitiba, Brazil; ^3^Department of Endocrinology, University of Naples Federico II, Naples, Italy

**Keywords:** acromegaly, complications, hypertension, insulin resistance, endothelium, neoplasm, quality of life, fractures

Acromegaly is a chronic disease mostly caused by a growth hormone (GH)-secreting pituitary adenoma. Excessive GH and insulin-like growth factor I (IGF-I) secretion in acromegaly promote tissues overgrowth, appearance changes, musculoskeletal disorders, and metabolic complications, which result in poor quality of life, increased mortality and decreased longevity when the disease is not adequately controlled (1, 2). Main causes of premature mortality in active acromegaly are cardiovascular, respiratory and neoplastic diseases (3–5), which are influenced by the concomitant presence of arterial hypertension, cardiomyopathy, arrhythmias, diabetes mellitus, and unfavorable lipid profile (2, 6).

The purpose of this special issue on health-related complications of acromegaly is to review the most common and jeopardizing comorbidities associated with the disease, since early diagnosis and optimal management of acromegaly and its comorbidities are critical to ensure best long-term outcomes.

Ramos-Leví and Marazuela reviewed the most relevant comorbidities related to cardiovascular system in acromegaly patients. They have highlighted that duration of GH hypersecretion, age, and body mass index (BMI) are key determinant factors for cardiovascular abnormalities. In addition, arterial hypertension is present in the majority of patients, especially in women, being characterized by elevated diastolic blood pressure and higher prevalence of non-dippers. The authors emphasize the importance of a lifelong treatment and control of hypertension in acromegaly to improve cardiovascular outcomes, and they have presented evidence that effective neurosurgery of the pituitary adenoma and medical therapies reduce left ventricular mass and improve cardiac function (7–9).

Puglisi et al. summarized the cardiometabolic consequences of acromegaly and its treatment, focusing on medical therapy with pasireotide. Although clinical studies demonstrated pasireotide LAR was more effective in acromegaly biochemical control than first generation somatostatin analogs (SSA), hyperglycaemia-related adverse events were common (10, 11). Mean HbA1c and fasting plasma glucose levels significantly increased (12). Baseline glucose status is a potential predictive factor for the development of hyperglycaemia during pasireotide LAR treatment. Therefore, before starting pasireotide, patients should undergo a glucose metabolism evaluation, and in case of glycaemic abnormalities, antidiabetic medication should be optimized.

Vila et al. discuss mechanisms leading to insulin resistance in acromegaly. An impairment of glucose metabolism is observed in over 50% of newly diagnosed patients. Insulin resistance plays an important role in the development of diabetes, hypertension, cardiovascular disease, sleep apnea, polycystic ovary syndrome, and can potentially contribute to cancer promotion. Acromegaly therapies might impact glucose metabolism on different ways. Successful surgery decreases plasma glucose (13, 14). The effects of SSAs on glucose metabolism depend on the status of glucose impairment before starting therapy: SSA may increase glucose levels in patients with normal or impaired glucose tolerance, and this effect might be abolished by metformin (15). In patients with diabetes, both impairment and improvement of glucose tolerance have been observed, in some cases with a need for optimization of diabetes therapy (15). Pasireotide strongly suppresses insulin secretion and interferes with incretins secretion, therefore hyperglycemia is observed in more than half of the patients (14). Pegvisomant shows a significant improvement in glucose metabolism and reduces the need for antidiabetic medications, meaning this drug is the most appropriate therapy in acromegaly patients with poorly controlled diabetes. Pegvisomant dose requirements depend on the severity of diabetes, and its positive effects on glucose metabolism are preserved in combined therapy with SSA (14, 16).

In their review,  Maffei et al. examined the controversial relationship between GH/IGF-I excess and the endothelium. In acromegaly, both GH and IGF-I might promote endothelial dysfunction and atherosclerosis through many different mechanisms (17). The role of lipid disorders, proinflammatory cytokines, and cell adhesion molecules has been studied without conclusive results. Recent study showed that coronary flow reserve (CFR), a marker of coronary microvascular function, was lower in acromegaly patients. An independent association between CFR and IGF-I suggests an important role of IGF-I in microvascular dysfunction in acromegaly that could be partially improved by disease control (18). Acromegaly patients do not always present with clear atherosclerotic damage and coronary heart disease.

Ruchala and Wolinski discussed the risk of malignant neoplasms in acromegaly. Among them, thyroid and colorectal cancer are most commonly associated with acromegaly. Meta-analysis confirmed increase in overall cancer and thyroid cancer incidence (19). According to studies published over the current decade cancer became the most common cause of mortality in this group (5). Differentiated thyroid cancer, in general, is characterized by relatively slow growth, low mortality and good prognosis. In authors' opinion thyroid ultrasound should be also performed at the baseline. Increased risk of these malignancies as well as low cost and invasiveness of screening test support their thesis.

In their review,Dworakowska and Grossman focused on the role of colon screening in preventing major acromegaly-related complications. They discussed disease pathophysiology especially in the context of its influence on colonic function and neoplasia. The reported prevalence of colonic polyps in patients with acromegaly has ranged from 6 to 30% for both adenomatous and non-adenomatous lesions, whereas that of colorectal carcinoma has varied between 4 and 10%. According to a large meta-analysis it is estimated that patients with acromegaly are 2–5 times more likely to develop colonic polyps than non-acromegalic controls (20). Authors anticipate that with such surveillance screening, the incidence of colorectal carcinoma in acromegaly may well-fall below that in the general population.

Since acromegaly is associated with impaired quality of life, Kunzler et al. assessed the effects of cognitive-behavioral therapy on acromegaly patients. The authors evaluated whether the “think healthy and feel the difference” technique in the short-term persists in the long-term, after the end of the treatment in a group of acromegalics (21). At the end of the study, there was improvement in the mental health of the intervention group compared to the controls. The effects of therapy were maintained at the 9 month follow-up. Authors concluded that a group cognitive-behavioral therapy can improve the quality of life of acromegalic patients in the short- and long-term.

Jawiarczyk-Przybyłowska et al. have investigated the potential association between vitamin D receptor (VDR) gene polymorphism and disease activity, bone density and quality, and fracture risk in patients with acromegaly. They found that FokI ff genotype might be related to better bone quality and effectiveness of acromegaly treatment. In addition, tt (TaqI), aa (ApaI), and bb (BsmI) genotypes were associated with better bone quality and microarchitecture. They also highlighted the importance of Trabecular Bone Score (TBS) as an useful tool for predicting fracture risk in acromegaly patients (22).

## Author Contributions

MB and AJ-P drafted the initial version of the manuscript. CB critically revised and completed the manuscript. All authors approved the final version to be published.

## Conflict of Interest

The authors declare that the research was conducted in the absence of any commercial or financial relationships that could be construed as a potential conflict of interest. The handling Editor declared a past co-authorship with the authors AC, MB, and CB.
